# Influence of panoramic image acquisition and digitization methods on fractal dimension measurement in panoramic radiographs

**DOI:** 10.3389/froh.2026.1877247

**Published:** 2026-07-02

**Authors:** Sana Faiza Fathani, Bramma Kiswanjaya, Syurri Innadinna Syahraini, Inka Saraswati, Aloysius Putut Wijanarko, Bayu Trinanda Putra, Menik Priaminiarti, Hanna H. Bachtiar-Iskandar

**Affiliations:** Department of Dentomaxillofacial Radiology, Faculty of Dentistry, Universitas Indonesia, Jakarta, Indonesia

**Keywords:** digital radiography, fractal dimension, image digitization, imaging variability, mandibular bone, panoramic radiography

## Abstract

**Objectives:**

Fractal dimension (FD) analysis is widely used as a quantitative radiographic method to characterize structural complexity in mandibular bone. However, FD measurements may be influenced by image acquisition and digitization procedures, potentially affecting comparability across imaging modalities. The effect of camera-digitized conventional panoramic radiographs on FD analysis remains insufficiently investigated. This study aimed to evaluate the influence of panoramic image acquisition methods on mandibular FD values by comparing camera-digitized conventional panoramic radiographs with digital panoramic radiographs.

**Methods:**

This cross-sectional study included 252 panoramic radiographs obtained from adults aged 25–75 years, consisting of 126 camera-digitized conventional and 126 digital panoramic radiographs. Fractal analysis was performed using the box-counting method on 11 mandibular regions of interest (ROIs) representing trabecular and cortical bone areas. FD measurements were analyzed according to imaging modality, bone microstructure type, age group, and sex. Interobserver and intraobserver reliability were evaluated using the intraclass correlation coefficient (ICC). Statistical analyses included multivariate analysis of variance (MANOVA), paired-samples t-tests, and Wilcoxon signed-rank tests, with a significance level of *p* < 0.05.

**Results:**

FD values differed significantly between imaging modalities across all ROIs (Wilks’ Lambda, *p* < 0.001), with higher FD values observed in camera-digitized conventional panoramic radiographs. Trabecular bone demonstrated significantly higher FD values than cortical bone (*p* < 0.001). Limited and inconsistent differences were observed across age and sex groups. Excellent intraobserver and interobserver agreement was achieved (ICC > 0.90). Therefore, the observed differences should be interpreted primarily as variability in FD measurements associated with imaging methods rather than direct evidence of differences in mandibular bone microarchitecture.

**Conclusion:**

Panoramic image acquisition and digitization methods significantly influence mandibular FD measurements. Camera-digitized conventional panoramic radiographs consistently demonstrated higher FD values than digital panoramic radiographs, indicating that FD measurements are sensitive to imaging-related variability. The observed FD variability likely reflects a combination of biological variation and technical influences associated with image acquisition, digitization, and image processing. These findings emphasize the need for standardized image acquisition and digitization protocols to improve the reliability, reproducibility, and comparability of FD analysis, particularly in retrospective studies using archived radiographs.

## 1 Introduction

Panoramic radiography is one of the most widely used imaging modalities in dentistry because it provides a comprehensive overview of the maxillofacial structures with relatively low radiation exposure, broad accessibility, and cost-effectiveness (1, 2). In addition to routine diagnostic evaluation, panoramic radiographs have increasingly been used for quantitative image analysis of mandibular bone structures, including assessment of trabecular complexity and cortical morphology (3, 4).

Fractal analysis (FA) is a mathematical image-processing method used to evaluate complex geometric structures that exhibit self-similarity across different scales (5). In dental radiology, FA has been widely applied to assess mandibular trabecular and cortical bone patterns on panoramic and periapical radiographs (3, 6). The resulting fractal dimension (FD) value provides a quantitative descriptor of structural complexity observed in radiographic bone patterns and has been investigated in various conditions, including osteoporosis, bruxism, temporomandibular disorders, inflammatory diseases, and systemic bone alterations (7–11). Compared with subjective visual interpretation, FD analysis offers a more objective and reproducible approach for evaluating radiographic bone patterns (3, 12). Several technical and biological factors may influence FD measurements. Previous studies demonstrated that region-of-interest (ROI) selection, image resolution, preprocessing methods, and radiographic exposure conditions can affect FD values (5, 13–15). Biological variables such as age, sex, bone remodeling activity, cortical thickness, and trabecular density may also contribute to variations in mandibular FD measurements (2, 16, 17). Consequently, interpretation of FD values requires careful consideration of both imaging-related and anatomical factors.

Digital panoramic radiography has largely replaced conventional film-based radiography because of its lower radiation dose, rapid image acquisition, image enhancement capability, and environmentally favorable workflow (18, 19). Nevertheless, Camera-digitized conventional panoramic radiographs are still used in many institutions and archived film images are frequently digitized for clinical documentation, retrospective studies, and image analysis purposes (20). Conventional radiographs may be digitized using scanners or digital cameras, both of which can introduce variations in image quality, contrast, distortion, and noise characteristics (20–23). Previous investigations comparing conventional and digitized radiographs have mainly focused on diagnostic accuracy and image quality assessment (21, 24–26). Studies evaluating the influence of image acquisition and digitization methods on quantitative radiographic analysis remain limited. In particular, the effect of camera-digitized conventional panoramic radiographs on FD measurements has not been sufficiently clarified. Because FD analysis is highly dependent on grayscale distribution and trabecular pattern representation, differences in detector characteristics, image processing, exposure parameters, and digitization artifacts may alter FD values and reduce comparability between imaging modalities (14, 15, 27).

Several studies have reported that image noise and radiographic processing may affect FD calculations (14, 27, 28). Furthermore, variability in digitization procedures, including camera exposure settings, positioning, resolution, and image standardization, may influence the reproducibility of quantitative image analysis (22, 23). Understanding the extent to which image acquisition methods influence FD measurements is important for interpreting results derived from retrospective radiographic archives and for improving methodological standardization in dental imaging research. Therefore, this study aimed to evaluate the influence of panoramic image acquisition methods on mandibular FD measurements by comparing camera-digitized conventional panoramic radiographs with digital panoramic radiographs. The present study specifically focuses on methodological variability associated with image acquisition and digitization rather than direct assessment of mandibular bone microarchitecture. FD values were analyzed in multiple mandibular cortical and trabecular regions according to imaging modality, bone microstructure type, age group, and sex. We hypothesized that different panoramic image acquisition methods would produce significantly different FD values.

## 2 Materials and methods

### 2.1 Study design and ethical approval

This cross-sectional comparative study was conducted at the Department of Dentomaxillofacial Radiology, Faculty of Dentistry, Universitas Indonesia, and the Dental and Oral Hospital, Universitas Indonesia, between September and December 2025. Panoramic radiographs obtained between 2017 and 2025 were retrospectively collected and analyzed. The study protocol was approved by the Dental Research Ethics Committee, Faculty of Dentistry, Universitas Indonesia (Protocol No. 011311025), and all procedures were performed in accordance with the Declaration of Helsinki. Because anonymized retrospective radiographic data were used, the requirement for individual informed consent was waived by the ethics committee.

### 2.2 Radiographic sample selection

A total of 252 panoramic radiographs from adults aged 25–75 years were included in this study, consisting of 126 camera-digitized conventional panoramic radiographs and 126 digital panoramic radiographs. The minimum sample size was calculated using G*Power software version 3.1.9.7 based on a previously reported medium effect size in mandibular fractal dimension analysis studies (4). Using a significance level of 0.05 and a statistical power of 95%, the minimum required sample size was estimated to be 210 radiographs. To compensate for potential exclusion due to image quality or incomplete data, the sample size was increased by approximately 20%, resulting in a final sample size of 252 panoramic radiographs.

The samples were categorized according to imaging modality, age group, sex, and bone microstructure type. Age was classified into five groups: 25–35, 36–45, 46–55, 56–65, and 66–75 years. Only radiographs with diagnostically acceptable image quality and clearly identifiable regions of interest (ROIs) were included. Radiographs presenting severe positioning errors, motion artifacts, distortion, extensive pathological lesions, or structural destruction affecting ROI interpretation were excluded from the analysis.

Two panoramic imaging systems manufactured by Yoshida (Tokyo, Japan) were used in this study. Camera-digitized conventional panoramic radiographs were acquired using exposure parameters of 76 kVp, 10 mA, and 16 s exposure time. The radiographic films were subsequently digitized using a digital camera (Canon PowerShot A2500; Canon Inc., Tokyo, Japan) and stored in JPEG format with standardized dimensions of 4608 × 3456 pixels and 24-bit depth. Digital panoramic radiographs were acquired using a photostimulable phosphor (PSP)-based digital system with exposure parameters ranging from 64 to 70 kVp, 3–8 mA, and 7.4 s exposure time. Digital images were retrieved using the manufacturer's software (i-Dixel; J. Morita Corp., Kyoto, Japan). To minimize variations during analysis, all radiographic images were resized to identical dimensions before fractal analysis was performed.

### 2.3 Fractal dimension analysis

**Figure 1:**
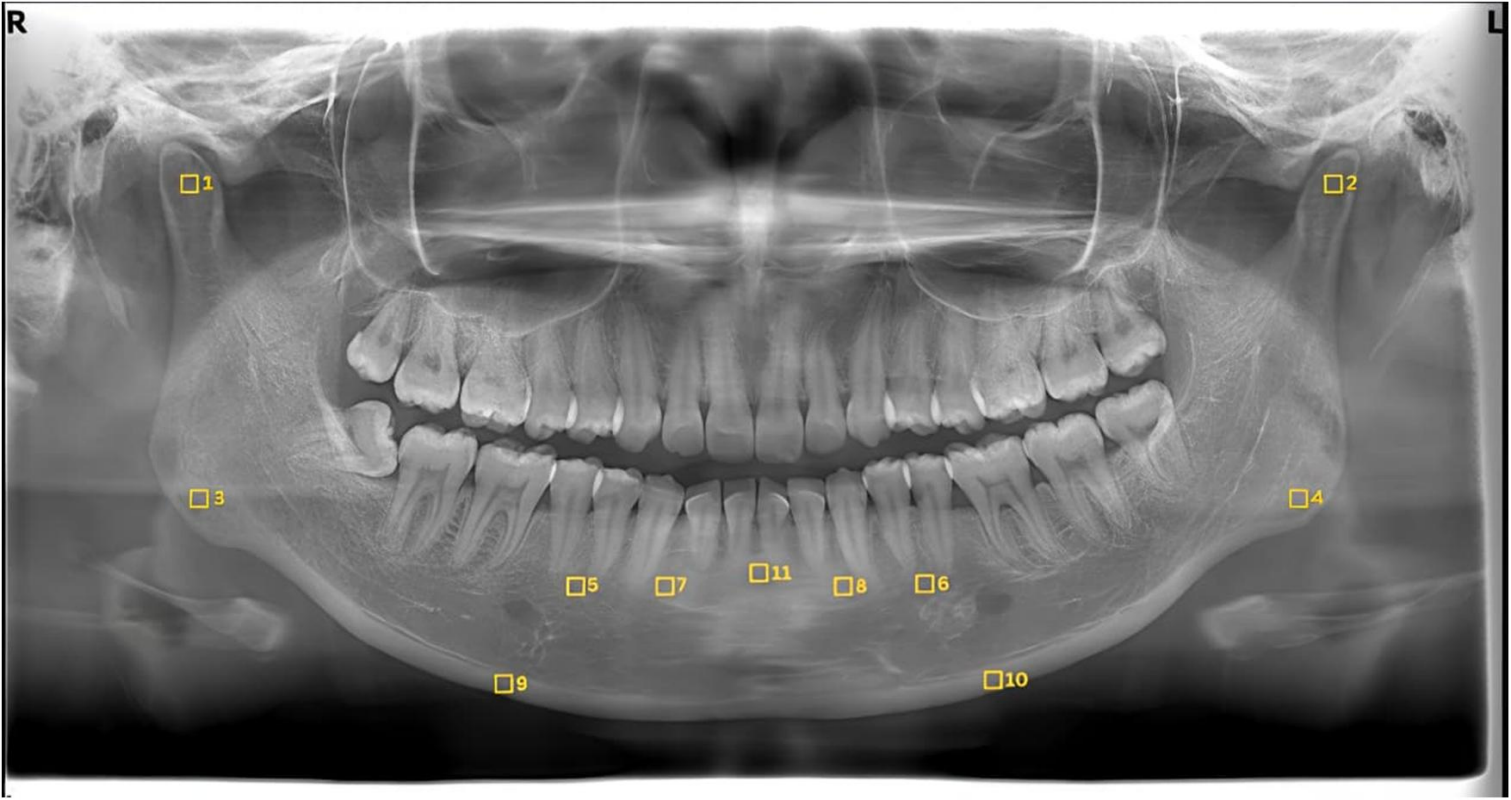
Regions of interest (ROIs) locations were selected to represent both trabecular and cortical mandibular bone regions while maintaining consistent ROI dimensions and anatomical reproducibility across subjects. ROI 1–2: mandibular condyle; ROI 3–4: mandibular angle; ROI 5–6: premolar region; ROI 7–8: canine region; ROI 9–10: basal cortical bone; ROI 11: mandibular incisor region.

The research design is shown in Figure 1, comprising 11 ROIs. In the trabecular bone area, there were 9 ROIs (mandibular condyle, mandibular angle, mesial apical canine of mandible, distal apical first premolar of mandible, and central apical incisor of mandible). In the cortical bone area, 2 ROIs (basal cortical bone of the mandible) were defined, one in each left and right region. ROI locations were selected based on previously published studies evaluating mandibular trabecular and cortical bone structures on panoramic radiographs (4, 9, 13, 15). The selected ROIs represented anatomically distinct regions with different structural characteristics while avoiding overlap with dental roots, anatomical superimpositions, cortical boundaries, and pathological lesions. To improve reproducibility and minimize measurement variability, all ROIs were standardized to identical dimensions (64 × 64 pixels) and positioned using consistent anatomical landmarks across all radiographs. FD assessment was conducted at 2,772 ROIs using the free software ImageJ, bundled with 64-bit Java 8 (available at https://imagej.nih.gov/ij/). After image acquisition, all images were stored in high-resolution JPEG format to preserve image quality while maintaining consistency across the dataset. To ensure uniformity, all images were resized to 4608 × 3456 pixels. After adjusting the images to meet the research criteria, the researchers downloaded them in JPEG format and performed fractal analysis via the box-counting method of White and Rudolph to obtain FD values (29). The results were recorded in Microsoft Excel and analyzed using IBM SPSS Statistics.

**Figure 2:**
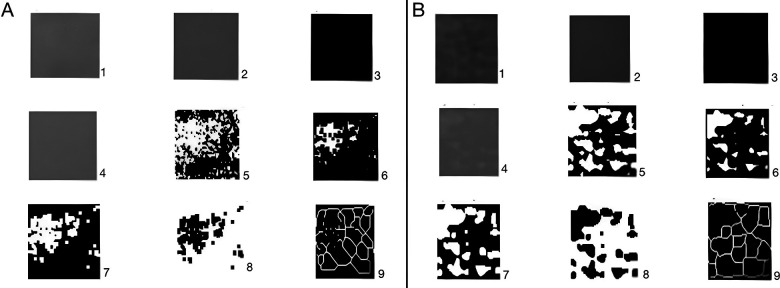
Workflow of fractal dimension analysis in camera-digitized conventional panoramic radiographs **(A)** and digital panoramic radiographs **(B)**, including ROI selection, image preprocessing, and skeletonization procedures prior to box-counting analysis.

The preprocessing procedure included ROI cropping and duplication, followed by removal of large-scale illumination variations using a Gaussian blur filter (sigma = 35 pixels; kernel size = 33 × 33). The processed image then underwent subtraction from the original image, addition of a gray value constant (128), binarization, erosion, dilation, image inversion, and skeletonization to preserve the main trabecular structures (29). The preprocessing workflow is illustrated in Figure 2. Following image preprocessing, FD values were calculated using the box-counting algorithm, one of the most commonly used methods because of its accessibility and ease of implementation (30–32). This method is based on gradual image coarsening, in which the original image represents the finest-scale approximation, while lower-resolution approximations are used to generate a log-log relationship between surface area and window size.

### 2.4 Reliability assessment

FD measurements were independently performed by two observers experienced in dentomaxillofacial radiology. To assess intraobserver and interobserver agreement, 20% of the samples were randomly re-evaluated after a two-week interval. Reliability was analyzed using the intraclass correlation coefficient (ICC). Of the 252 total samples, 50 radiographs (20%), comprising 25 camera-digitized conventional and 25 digital panoramic radiographs, were selected for intraobserver and interobserver reliability assessment. Reliability testing was conducted using measurements from ROI 5.

### 2.5 Statistical analysis

Statistical analyses were performed using IBM SPSS Statistics version 29.0 (IBM Corp., Armonk, NY, USA). Descriptive statistics were presented as mean ± standard deviation (SD). Data distribution was assessed using the Kolmogorov–Smirnov test. Comparisons of FD values according to imaging modality, age group, and sex were performed using multivariate analysis of variance (MANOVA). MANOVA was selected as the primary inferential approach to reduce the risk of inflated type I error associated with multiple ROI comparisons. When statistically significant differences were identified, univariate analyses and *post hoc* tests were subsequently conducted. Comparisons between cortical and trabecular bone FD values were performed using paired-samples t-tests or Wilcoxon signed-rank tests, depending on data distribution. Because cortical and trabecular FD measurements were derived from the same participant and the same panoramic radiograph, the observations were considered paired rather than independent. Statistical significance was set at *p* < 0.05.

## 3 Results

### 3.1 Reliability analysis

The results demonstrated excellent reliability, with interobserver and intraobserver ICC mean values of 0.935 and 0.931, respectively. The highest agreement was observed between the first examiner's initial measurement and the second examiner's second measurement (ICC = 0.976), whereas the lowest agreement was observed between the first examiner's second measurement and the second examiner's initial measurement (ICC = 0.899). Overall, the reliability analysis demonstrated excellent agreement for FD measurements, indicating high reproducibility between observers and repeated assessments.

### 3.2 Distribution of fractal dimension values

The distribution of FD values according to imaging modality, age group, and sex is summarized in Table 1. Across all ROIs, camera-digitized conventional panoramic radiographs generally demonstrated higher FD values than digital panoramic radiographs. In the digital imaging group, the highest mean FD value was observed in ROI 4 among males aged 46–55 years (1.568 ± 0.050), whereas the lowest value was identified in ROI 9 among females aged 36–45 years (1.312 ± 0.088). In the camera-digitized conventional imaging group, the highest mean FD value was found in ROI 1 among males aged 25–35 years (1.659 ± 0.061), while the lowest value was observed in ROI 11 among males aged 66–75 years (1.459 ± 0.107).

**Table 1:** Distribution of fractal dimension values according to imaging modality, Age group, and Sex.

ROI	Age range	Mean FD Value			
		Camera-digitized Conventional Panoramic Radiographs		Digital Panoramic Radiographs	
		Male	Female	Male	Female
1	Age 25–35	1,659 ± 0,061	1,604 ± 0,074	1,541 ± 0,061	1,484 ± 0,092
	Age 36–45	1,640 ± 0,068	1,587 ± 0,100	1,483 ± 0,117	1,481 ± 0,077
	Age 46–55	1,637 ± 0,094	1,569 ± 0,127	1,528 ± 0,071	1,496 ± 0,076
	Age 56–65	1,620 ± 0,035	1,583 ± 0,096	1,505 ± 0,055	1,503 ± 0,082
	Age 66–75	1,608 ± 0,074	1,621 ± 0,092	1,494 ± 0,082	1,480 ± 0,100
2	Age 25–35	1,610 ± 0,080	1,608 ± 0,088	1,519 ± 0,064	1,489 ± 0,076
	Age 36–45	1,626 ± 0,048	1,615 ± 0,072	1,471 ± 0,080	1,481 ± 0,085
	Age 46–55	1,637 ± 0,048	1,598 ± 0,091	1,511 ± 0,079	1,477 ± 0,058
	Age 56–65	1,583 ± 0,105	1,586 ± 0,084	1,525 ± 0,064	1,469 ± 0,074
	Age 66–75	1,625 ± 0,044	1,604 ± 0,070	1,482 ± 0,093	1,448 ± 0,127
3	Age 25–35	1,597 ± 0,072	1,598 ± 0,112	1,484 ± 0,079	1,459 ± 0,107
	Age 36–45	1,641 ± 0,055	1,595 ± 0,072	1,477 ± 0,087	1,482 ± 0,098
	Age 46–55	1,634 ± 0,043	1,565 ± 0,105	1,526 ± 0,055	1,486 ± 0,081
	Age 56–65	1,562 ± 0,128	1,562 ± 0,088	1,523 ± 0,090	1,509 ± 0,087
	Age 66–75	1,600 ± 0,125	1,606 ± 0,082	1,479 ± 0,132	1,485 ± 0,073
4	Age 25–35	1,585 ± 0,069	1,573 ± 0,086	1,496 ± 0,089	1,471 ± 0,123
	Age 36–45	1,587 ± 0,074	1,598 ± 0,082	1,489 ± 0,088	1,518 ± 0,070
	Age 46–55	1,606 ± 0,064	1,607 ± 0,071	1,568 ± 0,050	1,517 ± 0,093
	Age 56–65	1,563 ± 0,083	1,577 ± 0,120	1,558 ± 0,075	1,527 ± 0,065
	Age 66–75	1,590 ± 0,096	1,629 ± 0,064	1,486 ± 0,122	1,446 ± 0,127
5	Age 25–35	1,549 ± 0,077	1,559 ± 0,080	1,518 ± 0,052	1,509 ± 0,062
	Age 36–45	1,588 ± 0,060	1,570 ± 0,089	1,462 ± 0,060	1,491 ± 0,061
	Age 46–55	1,544 ± 0,057	1,560 ± 0,059	1,499 ± 0,039	1,483 ± 0,063
	Age 56–65	1,553 ± 0,079	1,578 ± 0,072	1,496 ± 0,092	1,455 ± 0,087
	Age 66–75	1,612 ± 0,069	1,591 ± 0,068	1,485 ± 0,071	1,451 ± 0,087
6	Age 25–35	1,585 ± 0,064	1,570 ± 0,059	1,501 ± 0,070	1,487 ± 0,061
	Age 36–45	1,573 ± 0,107	1,564 ± 0,082	1,481 ± 0,078	1,424 ± 0,110
	Age 46–55	1,548 ± 0,110	1,561 ± 0,078	1,445 ± 0,083	1,502 ± 0,055
	Age 56–65	1,593 ± 0,067	1,569 ± 0,080	1,472 ± 0,066	1,483 ± 0,067
	Age 66–75	1,556 ± 0,067	1,528 ± 0,117	1,454 ± 0,088	1,507 ± 0,047
7	Age 25–35	1,568 ± 0,049	1,560 ± 0,081	1,498 ± 0,078	1,476 ± 0,083
	Age 36–45	1,509 ± 0,109	1,553 ± 0,080	1,435 ± 0,087	1,422 ± 0,099
	Age 46–55	1,536 ± 0,085	1,486 ± 0,126	1,485 ± 0,062	1,498 ± 0,050
	Age 56–65	1,561 ± 0,096	1,552 ± 0,075	1,522 ± 0,057	1,498 ± 0,054
	Age 66–75	1,518 ± 0,095	1,551 ± 0,085	1,483 ± 0,072	1,446 ± 0,106
8	Age 25–35	1,534 ± 0,080	1,541 ± 0,073	1,504 ± 0,070	1,464 ± 0,061
	Age 36–45	1,561 ± 0,113	1,496 ± 0,096	1,449 ± 0,065	1,465 ± 0,078
	Age 46–55	1,563 ± 0,057	1,527 ± 0,091	1,497 ± 0,065	1,492 ± 0,085
	Age 56–65	1,569 ± 0,101	1,548 ± 0,072	1,519 ± 0,060	1,498 ± 0,080
	Age 66–75	1,560 ± 0,121	1,504 ± 0,113	1,489 ± 0,082	1,504 ± 0,055
9	Age 25–35	1,506 ± 0,073	1,499 ± 0,087	1,383 ± 0,103	1,362 ± 0,081
	Age 36–45	1,549 ± 0,052	1,516 ± 0,098	1,339 ± 0,066	1,312 ± 0,088
	Age 46–55	1,547 ± 0,037	1,534 ± 0,073	1,434 ± 0,087	1,400 ± 0,070
	Age 56–65	1,504 ± 0,072	1,541 ± 0,088	1,455 ± 0,125	1,407 ± 0,072
	Age 66–75	1,532 ± 0,064	1,558 ± 0,053	1,369 ± 0,101	1,396 ± 0,099
10	Age 25–35	1,515 ± 0,072	1,520 ± 0,064	1,368 ± 0,077	1,329 ± 0,065
	Age 36–45	1,537 ± 0,051	1,517 ± 0,062	1,336 ± 0,074	1,312 ± 0,080
	Age 46–55	1,538 ± 0,059	1,524 ± 0,065	1,403 ± 0,139	1,417 ± 0,067
	Age 56–65	1,501 ± 0,086	1,541 ± 0,085	1,420 ± 0,090	1,417 ± 0,087
	Age 66–75	1,531 ± 0,070	1,571 ± 0,067	1,379 ± 0,129	1,361 ± 0,107
11	Age 25–35	1,516 ± 0,107	1,466 ± 0,109	1,511 ± 0,065	1,436 ± 0,069
	Age 36–45	1,513 ± 0,105	1,505 ± 0,081	1,441 ± 0,096	1,407 ± 0,113
	Age 46–55	1,521 ± 0,055	1,541 ± 0,068	1,475 ± 0,074	1,479 ± 0,087
	Age 56–65	1,551 ± 0,061	1,461 ± 0,134	1,547 ± 0,041	1,490 ± 0,069
	Age 66–75	1,459 ± 0,107	1,484 ± 0,131	1,483 ± 0,062	1,477 ± 0,068

### 3.3 Comparison of FD values according to imaging modality

MANOVA demonstrated a statistically significant overall difference in FD values between imaging modalities across the 11 ROIs (Wilks' Lambda, *p* < 0.001). Camera-digitized conventional panoramic radiographs consistently demonstrated higher FD values than digital panoramic radiographs. Detailed comparisons according to imaging modality, age group, sex, and bone microstructure type are presented in Table 2. Subsequent univariate analyses revealed statistically significant differences in all ROIs (*p* < 0.05), with effect sizes ranging from small to large (partial *η*^2^ = 0.020–0.463). The largest differences between imaging modalities were observed in the cortical bone ROIs (ROI 9 and ROI 10). Because exposure parameters in the Camera-digitized conventional imaging group remained constant, correlation analysis between exposure settings and FD values could not be performed.

**Table 2:** Comparison of fractal dimension values according to imaging modality, Age group, Sex, and bone microstructure type.

Manova Test		ROI 1	ROI 2	ROI 3	ROI 4	ROI 5	ROI 6	ROI 7	ROI 8	ROI 9	ROI 10	ROI 11
Panoramic radiographs (Mean ± SD)	Digital	1,500 ± 0,082	1,488 ± 0,077	1,491 ± 0,089	1,507 ± 0,097	1,485 ± 0,070	1,477 ± 0,075	1,477 ± 0,080	1,488 ± 0,071	1,386 ± 0,096	1,375 ± 0,098	1,476 ± 0,083
	Camera-Digitized Conventional	1,613 ± 0,088	1,609 ± 0,075	1,596 ± 0,091	1,592 ± 0,081	1,569 ± 0,071	1,564 ± 0,086	1,539 ± 0,091	1,538 ± 0,093	1,529 ± 0,072	1,531 ± 0,069	1,502 ± 0,102
	*P*-value < 0,001											
	Univariate analysis	<0,001	<0,001	<0,001	<0,001	<0,001	<0,001	<0,001	<0,001	<0,001	<0,001	0,026
Age group (Mean ± SD)	Age 25–35	1,573 ± 0,095	1,557 ± 0,092	1,535 ± 0,111	1,532 ± 0,103	1,534 ± 0,070	1,536 ± 0,075	1,526 ± 0,082	1,512 ± 0,075	1,438 ± 0,107	1,434 ± 0,109	1,484 ± 0,093
	Age 36–45	1,550 ± 0,114	1,550 ± 0,101	1,551 ± 0,106	1,548 ± 0,090	1,528 ± 0,085	1,513 ± 0,110	1,480 ± 0,106	1,494 ± 0,098	1,432 ± 0,129	1,429 ± 0,122	1,468 ± 0,106
	Age 46–55	1,557 ± 0,107	1,556 ± 0,095	1,552 ± 0,092	1,575 ± 0,079	1,522 ± 0,063	1,516 ± 0,092	1,500 ± 0,087	1,519 ± 0,079	1,479 ± 0,093	1,472 ± 0,104	1,505 ± 0,076
	Age 56–65	1,551 ± 0,086	1,540 ± 0,093	1,539 ± 0,098	1,556 ± 0,088	1,520 ± 0,094	1,527 ± 0,086	1,533 ± 0,074	1,533 ± 0,080	1,477 ± 0,104	1,470 ± 0,100	1,511 ± 0,091
	Age 66–75	1,550 ± 0,108	1,536 ± 0,107	1,542 ± 0,117	1,539 ± 0,127	1,532 ± 0,098	1,508 ± 0,092	1,501 ± 0,096	1,510 ± 0,095	1,464 ± 0,116	1,462 ± 0,134	1,478 ± 0,095
	*P*-value 0,035											
	Univariate Analysis	0,758	0,783	0,906	0,218	0,901	0,537	0,024	0,256	0,092	0,171	0,111
Gender (Mean ± SD)	Male	1,569 ± 0,098	1,555 ± 0,093	1,549 ± 0,106	1,550 ± 0,091	1,527 ± 0,077	1,519 ± 0,095	1,510 ± 0,086	1,522 ± 0,088	1,457 ± 0,109	1,447 ± 0,114	1,502 ± 0,084
	Female	1,544 ± 0,105	1,541 ± 0,101	1,538 ± 0,103	1,550 ± 0,106	1,528 ± 0,087	1,522 ± 0,088	1,507 ± 0,095	1,505 ± 0,084	1,458 ± 0,113	1,458 ± 0,116	1,476 ± 0,100
	*P*-value 0,084											
	Univariate Analysis	0,054	0,249	0,392	0,991	0,958	0,779	0,079	0,134	0,908	0,485	0,025

Wilcoxon Test		Mean ± SD	*P*-value
Bone Microstructure Type	Cortical Bone	1,455 ± 0,105	< 0,001
	Trabecular Bone	1,528 ± 0,060	

### 3.4 Comparison of FD values according to bone microstructure type

Overall, trabecular bone demonstrated significantly higher FD values than cortical bone. The mean FD value for trabecular bone was 1.528 ± 0.060, whereas cortical bone demonstrated a mean FD value of 1.455 ± 0.105. The difference was statistically significant (*p* < 0.001). When analyzed separately according to imaging modality, trabecular bone also demonstrated significantly higher FD values than cortical bone in both the digital and camera-digitized conventional imaging groups (*p* < 0.001).

### 3.5 Comparison of FD values according to sex

No statistically significant overall difference in FD values between male and female participants was identified across the 11 rois (Wilks' Lambda, *p* = 0.084). However, univariate analysis demonstrated a statistically significant difference in ROI 11, where males showed slightly higher FD values than females (*p* = 0.025). When analyzed separately according to imaging modality, inconsistent findings were observed. In the digital imaging group, significant differences were identified in ROI 2 and ROI 11, whereas in the camera-digitized conventional imaging group, significant differences were observed in ROI 1 and ROI 8. The subgroup analyses performed separately for each imaging modality are summarized in Table 3. Because the primary objective of this study concerned imaging modality-related variability, sex-related analyses should be considered secondary exploratory findings.

**Table 3:** Subgroup analysis of fractal dimension values according to imaging modality.

Manova Test		ROI 1	ROI 2	ROI 3	ROI 4	ROI 5	ROI 6	ROI 7	ROI 8	ROI 9	ROI 10	ROI 11
Age group	*P*-value: 0,005 ^a^;0,360^b^											
	Univariate Analysis	0,527^a^ 0,712^b^	0,321 ^a^ 0,496^b^	0,348 ^a^ 0,260^b^	0,013^a^ 0,267^b^	0,136 ^a^ 0,137^b^	0,481^a^ 0,409^b^	0,003^a^ 0,200^b^	0,097 ^a^ 0,768^b^	0,002^a^ 0,183^b^	<0,001^a^ 0,313^b^	0,002^a^ 0,374^b^
Gender	*P*-value: 0,327^a^;0,035^b^											
	Univariate Analysis	0,136^a^ 0,007^b^	0,033^a^ 0,282^b^	0,429^a^ 0,150^b^	0,195^a^ 0,432^b^	0,241^a^ 0,691^b^	0,477^a^ 0,353^b^	0,297^a^ 0,956^b^	0,647^a^ 0,043^b^	0,305^a^ 0,832^b^	0,536^a^ 0,356^b^	0,027^a^ 0,191^b^

Wilcoxon Test^a^	Paired Samples T-Test^b^	*P*-value	*P*-value
Bone Microstructure Type	<0,001	Bone Microstructure Type	<0,001

^a^Digital panoramic radiographs.^b^Camera-digitized conventional panoramic radiographs.

### 3.6 Comparison of FD values according to age group

MANOVA demonstrated a statistically significant overall difference in FD values among age groups across the 11 ROIs (Wilks' Lambda, *p* = 0.035). Subsequent univariate analysis identified a significant difference only in ROI 7 (*p* = 0.024), with the highest mean FD value observed in the 56–65 years age group. *post hoc* analysis demonstrated that the most significant difference was found between the 56–65 years and 36–45 years age groups. When analyzed separately according to imaging modality, the digital imaging group demonstrated statistically significant differences in several ROIs, particularly ROI 4, ROI 7, ROI 9, ROI 10, and ROI 11. In contrast, the camera-digitized conventional imaging group showed no statistically significant overall differences across age groups. Age-related analyses were performed as secondary exploratory evaluations and should be interpreted cautiously.

## 4 Discussion

This study demonstrated that panoramic image acquisition methods significantly influenced mandibular FD measurements, with camera-digitized conventional panoramic radiographs consistently showing higher FD values than digital panoramic radiographs across all ROIs. These findings support the hypothesis that image acquisition and digitization procedures may substantially affect FD measurements obtained from panoramic radiographs. Fractal dimension should be interpreted as an image-derived measure of structural complexity rather than a direct measurement of bone microarchitecture. Consequently, FD values may reflect both biological characteristics of bone and technical factors associated with image acquisition, digitization, and image processing. Previous studies have demonstrated that FD measurements are influenced not only by trabecular architecture itself, but also by image resolution, preprocessing methods, grayscale distribution, radiographic noise, and other imaging-related variables (3, 5, 13–15, 21, 22). Therefore, the differences observed between imaging modalities in the present study are more appropriately interpreted as variability in FD measurements associated with image acquisition and digitization procedures rather than direct evidence of differences in mandibular bone microarchitecture.

It is important to acknowledge that multiple technical variables differed simultaneously between the two imaging groups, including detector technology, image acquisition systems, exposure parameters, image format, digitization procedures, and potentially proprietary post-processing algorithms. Consequently, the present study cannot determine which individual technical factor was primarily responsible for the observed FD differences. The findings should therefore be interpreted as reflecting the combined influence of multiple imaging-related factors.

FD analysis has been widely used to evaluate trabecular bone complexity in panoramic and periapical radiographs because higher FD values are generally associated with greater structural complexity and irregularity of bone patterns (3, 22, 29, 31, 32). Fractal analysis has also been applied to evaluate radiographic bone changes associated with periodontal, peri-implant, and alveolar bone conditions, where FD values have been reported to reflect alterations in trabecular pattern complexity and bone remodeling processes (6, 8). However, previous studies have also demonstrated that FD measurements remain sensitive to imaging conditions, ROI selection, preprocessing workflows, and image quality characteristics, emphasizing the importance of methodological standardization when interpreting quantitative radiographic findings (5, 13–15, 27, 33). Consequently, differences observed in FD values may not exclusively reflect biological alterations in bone structure but may also be influenced by variability introduced during image acquisition, digitization, and image analysis procedures (5, 21–23, 27, 28, 33). In the present study, higher FD values observed in camera-digitized conventional radiographs may be associated with increased image noise, variations in grayscale intensity, and digitization artifacts generated during camera-based image acquisition. Image noise is known to influence FD calculations by altering pixel orientation and disrupting the representation of anatomical structures (27, 28). Pant reported that noisy digital images tend to produce artificially increased FD values because noise modifies local pixel distribution and surface irregularity (27). Similarly, Reiss et al. demonstrated that box-counting FD values increase logarithmically in the presence of image noise, potentially resulting in overestimation of structural complexity (28). These observations may explain the systematically higher FD values identified in the camera-digitized conventional imaging group in the present study.

Visual differences between imaging modalities were also observed during preprocessing stages, particularly after erosion, dilation, and skeletonization procedures. Camera-digitized conventional images demonstrated more irregular and discontinuous skeletal patterns compared with digital panoramic radiographs, despite using identical preprocessing protocols. These findings suggest that image acquisition methods may influence the preservation of trabecular continuity during preprocessing. Previous investigations have similarly reported that preprocessing sensitivity and ROI characteristics may substantially affect FD reproducibility (5, 13, 33). Although studies evaluating FD analysis in digitized radiographs remain limited, Oliveira et al. compared FD values obtained from digital and digitized radiographs and reported no statistically significant differences between modalities (34). However, their study used scanner-based digitization, whereas the present study used camera-based digitization, which may produce different image noise characteristics and signal-to-noise ratios (22, 23). Davidson et al. and Sistrom and Gay also demonstrated that image quality and noise characteristics vary considerably among digitization devices, potentially influencing quantitative image analysis (22, 23). Recent studies have further demonstrated that ROI orientation, preprocessing workflows, and image standardization procedures can significantly influence FD measurements even when identical box-counting algorithms are used (14, 15, 35). Amuk et al. reported that variations in radiographic technical parameters may affect FD values depending on the imaging modality and acquisition conditions, highlighting the sensitivity of FD analysis to image-related factors (14). Similarly, Pekince et al. demonstrated that ROI orientation may alter FD measurements, whereas Boztaş Demir et al. emphasized the importance of methodological standardization when evaluating mandibular trabecular architecture using panoramic radiographs (15, 35).

Another important consideration is the variability inherent in camera-based digitization procedures. Factors such as ISO sensitivity, exposure time, image resolution, camera positioning, angulation, lighting conditions, and stabilization methods may influence image quality and introduce inconsistencies in grayscale representation (17, 22, 23, 36). In addition, JPEG compression may alter grayscale distribution and edge representation, potentially affecting image preprocessing outcomes and subsequent FD calculations. Differences in detector technology, image processing algorithms, exposure parameters, and image noise characteristics between conventional and digital imaging systems may further contribute to variability in FD measurements (14, 18, 19, 22, 23, 27, 28). Although all images in the present study were standardized to identical dimensions prior to analysis, complete standardization of retrospective digitization parameters could not be achieved. This limitation may partially explain the variability observed in FD measurements within the camera-digitized conventional group. Nevertheless, differences in detector characteristics, modulation transfer function, and image processing algorithms between conventional and digital systems may still contribute to FD variability (5, 18, 19).

The present study also demonstrated significantly higher FD values in trabecular bone compared with cortical bone. This finding is consistent with previous studies by Ersan et al. and Nursari et al., which reported lower FD values in cortical bone regions than in trabecular bone areas (7, 9). Trabecular bone contains a more heterogeneous and interconnected microarchitecture, whereas cortical bone demonstrates a relatively compact and homogeneous structure (10, 17, 27). Consequently, trabecular regions generally exhibit greater structural complexity and higher FD values.

As age- and sex-related analyses were included as secondary exploratory outcomes, these findings should be interpreted cautiously and in the context of the study's primary objective concerning imaging modality-related variability. Sex-related differences in FD values were limited and inconsistent across ROIs. Although isolated differences were identified in several regions, the overall MANOVA analysis showed no significant sex-related effect. These findings are consistent with previous studies reporting that mandibular FD measurements are only modestly influenced by sex and may vary according to anatomical location (4, 16). While differences in bone remodeling and hormonal status between males and females may affect skeletal structures, their influence on mandibular FD values appears less pronounced than the variability associated with imaging acquisition and digitization methods observed in the present study. Age-related differences were also limited, with only one ROI demonstrating a significant univariate difference despite a statistically significant overall MANOVA result. Previous studies have reported age-associated changes in trabecular organization and bone remodeling; however, these alterations may occur heterogeneously across mandibular regions (10, 17). The relatively small and inconsistent age-related differences observed in this study suggest that age had a lesser influence on FD measurements than imaging modality-related factors.

To the best of our knowledge, this study is among the first to specifically evaluate the influence of camera-digitized conventional panoramic radiographs on mandibular FD analysis. The findings highlight the importance of considering imaging modality and digitization procedures when interpreting FD measurements derived from retrospective radiographic archives. Although previous studies have supported the diagnostic reliability of digitized conventional radiographs for clinical interpretation (6, 20–26), the present findings suggest that quantitative image analysis techniques such as FD analysis may be more sensitive to digitization-related variability. Although FD analysis has been extensively applied in panoramic radiography, interpretation of FD values should consider the inherent limitations of two-dimensional imaging. Superimposition of anatomical structures, projection geometry, detector characteristics, and image processing algorithms may influence the representation of trabecular patterns. Three-dimensional imaging modalities such as CBCT may provide additional structural information; however, standardization of ROI selection, preprocessing protocols, and FD calculation methods remains essential regardless of imaging modality (9, 37).

Several limitations should be acknowledged. First, the retrospective design limited control over image acquisition, digitization, and preprocessing conditions. Second, multiple technical variables differed simultaneously between the camera-digitized conventional and digital imaging groups, including detector technology, exposure parameters, image format, noise characteristics, and potential post-processing algorithms. These factors may alter the visual representation of bone patterns and consequently affect fractal analysis outcomes. Therefore, the observed differences in FD values between imaging modalities should be interpreted with caution, as they may partially reflect technical characteristics of the imaging systems rather than exclusively representing biological differences in bone microarchitecture, preventing identification of the individual factor responsible for FD variability. Third, camera-based digitization parameters such as ISO sensitivity, exposure duration, lighting conditions, angulation, stabilization methods, and JPEG compression in camera digitization could not be fully standardized. Fourth, multiple ROI-specific comparisons may increase the risk of false-positive findings despite the use of MANOVA as the primary analytical framework. Finally, two-dimensional panoramic radiographs cannot fully represent the three-dimensional architecture of mandibular bone.

## 5 Conclusion

Panoramic image acquisition and digitization methods significantly influence mandibular FD measurements. Camera-digitized conventional panoramic radiographs consistently demonstrated higher FD values than digital panoramic radiographs, indicating that FD measurements are sensitive to imaging-related variability. Therefore, FD values should be interpreted as quantitative radiographic descriptors of structural complexity rather than direct representations of mandibular bone microarchitecture. The observed FD variability likely reflects a combination of biological variation and technical influences associated with image acquisition, digitization, and image processing. Trabecular bone exhibited higher FD values than cortical bone, whereas age- and sex-related differences were limited and inconsistent. These findings emphasize the need for standardized image acquisition and digitization protocols to improve the reliability, reproducibility, and comparability of FD analysis, particularly in retrospective studies using archived radiographs.

## Data Availability

The raw data supporting the conclusions of this article will be made available by the authors, without undue reservation.
